# Connecting Chromatin Modifying Factors to DNA Damage Response

**DOI:** 10.3390/ijms14022355

**Published:** 2013-01-24

**Authors:** Weiwei Lai, Hongde Li, Shuang Liu, Yongguang Tao

**Affiliations:** 1 Cancer Research Institute, Central South University, Changsha 410078, Hunan, China; E-Mails: weiwater235@tom.com (W.L.), azbyccx@163.com (H.L.); 2 Key Laboratory of Carcinogenesis and Cancer Invasion, Ministry of Education, Changsha 410078, Hunan, China; 3 Key Laboratory of Carcinogenesis Principal, Ministry of Health, Changsha 410078, Hunan, China; 4 Department of Dermatology, Xiangya Hospital, Central South University, Changsha 410008, Hunan, China; 5 Center for Medicine Science, Xiangya Hospital, Central South University, Changsha 410008, Hunan, China

**Keywords:** chromatin-remodeling factor, DNA damage response, signaling transduction pathway, cell cycle, protein interaction, protein modification, double strand breaks

## Abstract

Cells are constantly damaged by factors that can induce DNA damage. Eukaryotic cells must rapidly load DNA repair proteins onto damaged chromatin during the DNA damage response (DDR). Chromatin-remodeling complexes use the energy from ATP hydrolysis to remodel nucleosomes and have well-established functions in transcription. Emerging lines of evidence indicate that chromatin-remodeling complexes are important and may remodel nucleosomes during DNA damage repair. New studies also reveal that ATP-dependent chromatin remodeling is involved in cell cycle progression, signal transduction pathways, and interaction and modification of DDR-related proteins that are specifically and intimately connected with the process of DNA damage. This article summarizes the recent advances in our understanding of the interplay between chromatin remodeling and DNA damage response.

## 1. Introduction

Cells from yeast to mammals are constantly damaged by different factors [1]. Chromatin-remodeling factors are highly conserved, and the modification of chromatin structure during the DNA damage response (DDR) is common to yeast, *Droshophila*, mice and humans. Mammalian cells must rapidly load DNA repair proteins onto damaged chromatin in response to DNA damage. Chromatin-remodeling complexes use the energy from ATP hydrolysis to remodel nucleosomes and have well-established functions in transcription [2]. Moreover, chromatin-remodeling factors may remodel nucleosomes during DNA damage repair.

Chromatin remodeling refers to numerous changes in nucleosomal organization based on changes in DNA-histone contacts. The ATP-dependent chromatin-remodeling machineries are large multiprotein complexes containing a catalytic subunit that belongs to the SWI2/SNF2 superfamily of ATPases. ATP-dependent chromatin remodeling is one of the fundamental mechanisms used by cells to relax chromatin in DNA repair [3–5]. The superfamily includes five classes of chromatin-remodeling complexes: SWI/SNF, NuRD/Mi-2, ISWI, CHD and INO80 [6–10]. Numerous chromatin-remodeling factors, especially those from the INO80 and SWI2 subfamilies, are recruited to double strand breaks (DSBs) in response to DDR.

Environmental stress, such as ionizing radiation, and endogenous events, such as replication fork collapse and antigen receptor gene rearrangement, can all induce DSBs. In response to DSB lesions, cells rapidly activate a dedicated signal transduction network to convey the damage signal to many types of cellular machinery that are involved in different processes, including transcriptional regulation, cell cycle regulation, apoptosis and DNA repair. In eukaryotic cells, two conserved signaling pathways exist for DSB repair. Homologous recombination (HR) relies on the transfer of genetic information of a homologous molecule to the site of damage, whereas nonhomologous end joining (NHEJ) mediates DNA end ligation without recombination or sequence homology requirements [11–13]. In this review, we examine the recent advances in elucidating how chromatin-remodeling factors are recruited to DSB sites and function directly in response to DDR signals.

## 2. Signal Transduction Pathways in DNA Damage Response Communicate with Chromatin-Remodeling Factors

The INO80 (inositol requiring 80) subfamily is the one most recently elucidated, and includes the SWI/SNF family of chromatin-remodeling factors. The chromatin-remodeling complexes of the INO80 subfamily are: Tip60, INO80, snf2-related activator protein (SRCAP), TRAP, MRG15, p400 and H2AZ [6,9].

**Figure 1:**
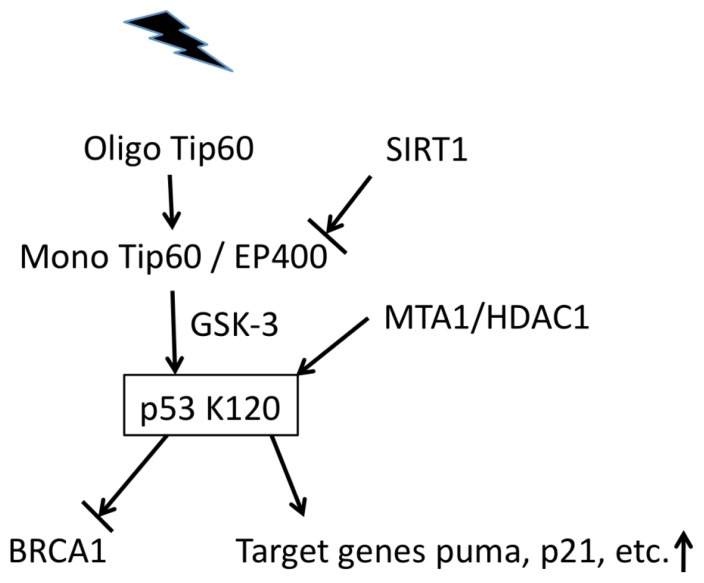
The p53 signaling pathway interacts with chromatin-remodeling factors in response to DNA damage. In response to DNA damage, Tip60 switches from an oligomer to a monomer, at which point it interacts with EP400. Glycogen synthase kinase-3 (GSK-3) is involved in the process of p53 activation by acetylation; in turn, activated p53 inhibits tumor suppressor BRCA1 (breast cancer type 1 susceptibility protein) and induces the p53 target gene PUMA, also known as p21. SIRT1 (sirtuin 1) inhibits the activity of Tip60 while MTA1 (metastasis-associated proteins 1)/HDAC1 (histone deacetylase 1) induces p53 activity.

The p53 signaling pathway responds to various cellular stress signals, including DNA damage, by regulating key processes, such as DNA repair, through its role as a key transcription factor [14]. Additionally, the p53 signaling pathway is also associated with chromatin structure changes that mainly involve Tip60 following DNA damage (Figure 1).

Tip60 is a ubiquitously expressed acetyltransferase. It is an essential activator in multiple signaling pathways, including chromatin remodeling, and plays a critical role in DSB repair [15,16]. Tip60 functions as an upstream component of UV DDR and enables UV-induced DDR signaling even in the absence of p53, whereas preaccumulated p53 suppresses UV-induced DDR by reducing the levels of BRCA1 (breast cancer type 1 susceptibility protein) that repairs double-strand breaks in DNA [17,18].

During normal cell proliferation, most Tip60 proteins form inactive oligomers in an unacetylated state. In response to DNA damage, Tip60 switches to its activated monomeric form after autoacetylation. Activated Tip60 then acetylates p53 at the Lys120 site that requires glycogen synthase kinase-3 (GSK-3) activity, promoting apoptosis and inducing p53 target genes such as PUMA. The HDAC sirtuin 1 (SIRT1), a negative regulator of Tip60, deacetylates Tip60, thereby regulating the level of activated Tip60 [18,19] (Figure 1).

The histone acetyl transferase Tip60 (HTATIP) complex, containing Tip60 and the E1A-associated p400 protein (EP400), is required for DNA damage-induced apoptosis. However, Tip60 and p400 have opposite effects on p21 expression in the absence of DNA damage. Interestingly, this change depends on the inhibition of Tip60 function by p400, a property that is abolished after DDR [20]. Moreover, the p400/Tip60 ratio controls the oncogene-induced DDR [21].

MTA1, a transcriptional coregulator on its target chromatin, is required for DSB repair. Moreover, MTA1 controls p53 stability and consequently regulates p53-dependent transcription of p53R2 ribonucleotide reductase, a gene responsible for supplying nucleotides for DNA repair following DNA damage and cell cycle checkpoints [22,23]. During UV treatment, MTA1 is required for the activation of the ATR-Claspin-Chk1 and ATR-H2AX pathways [24]. Furthermore, MTA1 is a p53-independent transcriptional corepressor of p21 (WAF1), and this mechanism involves the direct recruitment of the MTA1-histone deacetylase 2 (HDAC2) complexes to the p21 (WAF1)-proliferating cell nuclear antigen (PCNA) pathway [25]. Because more than 36 amino acids of p53 are modified by phosphorylation, ubiquitination, acetylation, and other posttranslational modifications [14], it remains unclear whether chromatin-remodeling factors impact p53 modification directly.

Members of the phosphatidylinositol 3-kinase-related kinase (PIKK) family, including the ATM, DNA-PKcs, ATR, and TRRAP proteins, function in signal transduction pathways that activate the DNA damage response. Cells depleted of tra1 activate Chk1 normally and are checkpoint proficient. The changed division response (Cdr) kinases Cdr1 and Cdr2, negative regulators of Wee1, are required for the Tra1-dependent alterations to Wee1 function [26].

Numerous chromatin-remodeling factors that are involved in DNA methylation and demethylation also contribute to DDR. In the thymus, genotoxic stress exposure decreases DNA methylation globally after the reduction of enzymes involved in DNA methylation such as DNA methyltransferase 1 (DNMT1), DNMT3a, DNMT3b and methyl-binding proteins MeCP2 and MBD2 [27]. Interestingly, DNA repair enzyme thymine DNA glycosylase (TDG) interacts with the damage response protein growth arrest and DNA damage-inducible protein 45 α (GADD45a), promoting, in its turn, DNA demethylation, which is essential for mammalian development [28]. TDG also contributes to the maintenance of active and bivalent chromatin throughout cell differentiation, facilitating the assembly of the chromatin modifying complex and initiating DNA repair mechanisms such as base excision repair [29]. However, it remains unclear which chromatin-remodeling factors are involved in the process of DDR and the mechanisms by which they are involved. Additionally, it is unclear whether DNA methylation and demethylation are directly associated with DDR.

## 3. Interplay of Chromatin Remodeling and DDR during Cell Cycle Progression

Cell cycle checkpoint activation is the initial response to DNA damage, delaying cell cycle progression until genome integrity is restored. The cell cycle is divided into four distinct phases, including the G1 phase, S phase (synthesis), G2 phase (collectively known as interphase) and M phase (mitosis). Chromatin-remodeling factors are involved throughout the process, while the G2/M checkpoint is more sensitive to DDR than the other phases. DDR could stall the cell cycle before the damaged DNA and chromatin is passed on to daughter cells. HR acts mainly in the S and G2 phases of cell cycle.

The nucleosome remodeling and histone deacetylase (NuRD) complex uniquely couples two chromatin-directed enzymatic functions: ATP-dependent nucleosome remodeling and histone deacetylation. The NuRD complex is composed of the chromatin-remodeling subunit CHD4/3 (chromodomain helicase DNA-binding protein 4 and 3), HDAC1/2 (histone deacetylase 1 and 2), RbAp (retinoblastoma-associated protein 46 and 48), p66a, p66b, MBD (methyl-CpG-binding domain-containing protein 2 and 3), and MTA1-3 (metastasis-associated proteins 1, 2, and 3) [8,30]. The NuRD subunits CHD4, CHD3, MTA1, and MTA2 all showed a dramatic increase in chromatin binding in response to the irradiation response (IR) in the early stage of DDR [31]. CHD4 is enriched at DNA damage sites as a NuRD complex after IR in a poly (ADP-ribose)-dependent manner. Moreover, CHD4 functions as a key regulator of the G1/S cell cycle transition by p53 deacetylation [32].

**Figure 2:**
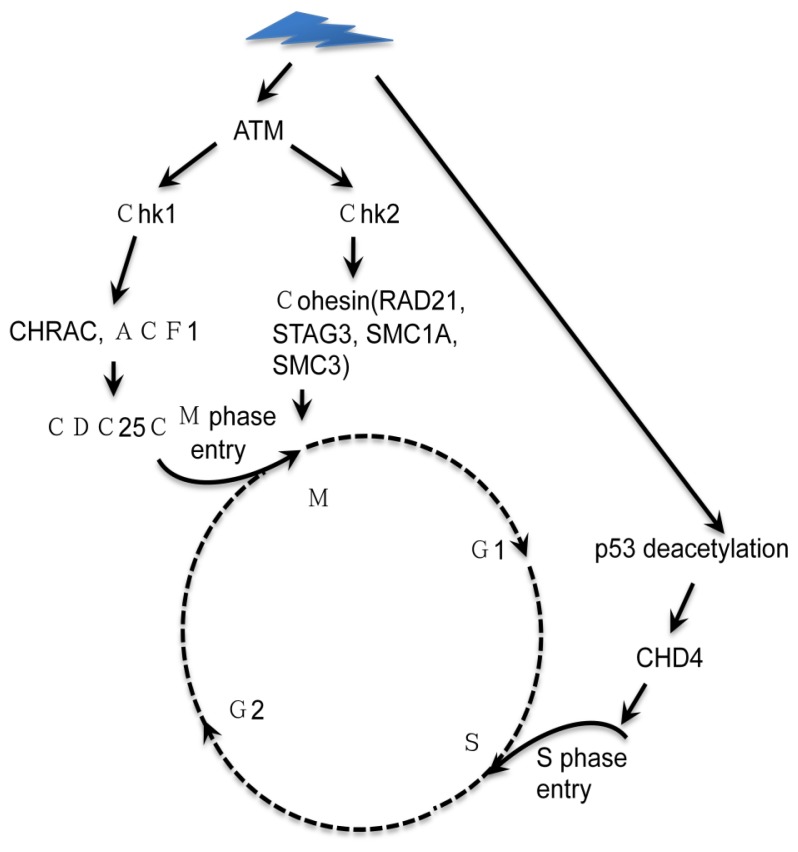
Chromatin-remodeling factors affect the cell cycle. On one hand, damage signals induce deacetylation of p53; in turn, the chromodomain helicase DNA-binding protein 4 (CHD4) prevents entry into S phase. On the other hand, CHRAC (CHRomatin accessibility complex), ACF1 (ATP-dependent chromatin assembly and remodeling factor 1) and cohesin are affected by checkpoint kinases after the ATM signaling pathway is activated by DNA damage, which prevents the activation of CDC25 and the transition from G2 to M.

The G2 DNA damage checkpoint inhibits Cdc2 and mitotic entry through the dual regulation of Wee1 and Cdc25 by the Chk1 effector kinase. Transformation/transcription domain-associated protein (TRRAP) is a component of several multiprotein acetyltransferase complexes implicated in both transcriptional regulation and DNA repair. In fission yeast, a mutation in tra1, which encodes one of two homologs of TRRAP, an ATM/R-related pseudokinase that scaffolds several HAT complexes [26], irradiation (IR) induces H2AacK5 reduction and synaptonemal complex (SC) disassembly in a Tip60-dependent manner [33]. The nucleosome sliding factors chromatin accessibility complex (CHRAC) and ATP-dependent chromatin assembly and remodeling factor (ACF) are required for a tight checkpoint, and they are induced upon replication fork collapse in response to UV and X rays [34], indicating a critical role for ACF1 in the G2/M damage checkpoint (Figure 2).

Cohesin, which functions as a special chromatin-remodeling factor, is established during S phase as DNA is replicated and is lost when chromosomes segregate during mitosis and meiosis. Meiosis is resistant to DDR due to chromosome structure. Cohesin containing the proteins RAD21, STAG3, structural maintenance of chromosomes 1A (SMC1A) and SMC3, plays numerous key roles in DDR [35–38]. During M phase, cohesins are implicated in mitotic spindle function through SMC1A and RAD21 (and its homolog REC8), which are localized to the centrosome along with SMC3, which binds and stabilizes microtubules [35]. Recently, cohesin was generated by an Eco1-dependent but replication-independent mechanism in response to DSBs in G2/M, and the DNA damage checkpoint mediated by Eco1 provided genome-wide protection of chromosome integrity [39]. Sister chromatid cohesion is essential for both chromosome segregation and DSB repair and is controlled by the DNA damage response and cohesin-regulating factors [40]. Inactivation of cohesin impairs recruitment of 53BP1 (p53-binding protein 1), an important mediator of the DDR, to IRIFs and leads to defects in activation of the checkpoint kinase Chk2 [41]. Four ISWI/cohesin family factors and four SWI/SNF family chromatin-remodeling factors are implicated in the UV damage response that depends on developmental stage [42]. These findings indicate a complex role for cohesin in gene expression and DNA repair, especially during G2/M phase. Whether other chromatin-remodeling factors are involved requires further investigation.

## 4. Interaction between DDR-Related Proteins and Chromatin-Remodeling Factor

Interaction between DDR-related proteins is a hallmark of the cellular response to DNA damage. Several chromatin-remodeling factors form a complex with DDR-related proteins in response to DNA damage. Scaffold matrix attachment region 1 (SMAR1) binds other SMAR1 elements along with HDAC1 and p53, forming a repressor complex to downregulate transcription. However, SMAR1 increases p53 acetylation and BAX and PUMA transcription instead of interacting with MAR elements to induce apoptosis in response to DNA damage [43].

Tip60 plays a critical role in the DNA damage response by interacting with other proteins beyond the p53 signaling pathway, as discussed above. Tip60 also interacts with the transcription factor E2F1 to promote its acetylation in response to DNA damage by cisplatin [44]. The ataxia telangiectasia mutant (ATM) protein kinase, which phosphorylates proteins involved in cell-cycle checkpoints and DNA repair, is regulated by the Tip60 HAT in response to DNA damage [45]. Moreover, direct interaction between the chromodomain of Tip60 and histone H3 trimethylated on lysine 9 (H3K9me3) at DSBs activates the acetyltransferase activity of Tip60 [9,15,46]. PIKK proteins contain a conserved C-terminal FAT/kinase domain/FATC domain structure, and the FATC domain of ATM mediates the interaction between ATM and Tip60. The conserved FATC domain of PIKK proteins may therefore function as a binding domain for the Tip60 histone acetyltransferase in response to DNA damage [47]. Tip60 haplo-insufficiency does not affect B-cell homeostasis but abrogates Myc-induced DDR [48]. IR could increase the interaction of MTA1 with ATR, resulting in stabilizing MTA1 in an ATR-dependent manner [24]. PML3, a PML isoform, specifically interacts with and recruits Tip60 to PML NBs. This interaction modulates NBs distribution and mobility of Tip60 in the DNA damage-repairing response [49].

**Figure 3:**
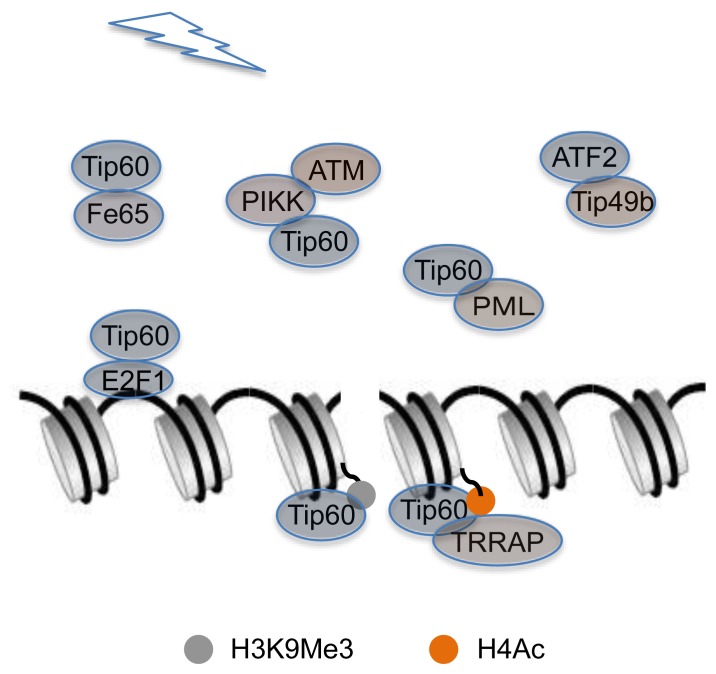
Interaction between DDR-related proteins and Tip49b or Tip60. In response to DNA damage, Tip49b interacts with ATF2. Meanwhile, Tip60 may interact with other DDR-related factors including Fe65 and PML. PIKK could bridge the interaction of ATM and Tip60. Moreover, Tip60 could be recruited to the DNA by interaction with transcription factor E2F1. Interestingly, Tip60 is directly targeted to the nucleosomes of DSBs by another chromatin-remodeling factor known as TRRAP and histone modifications such as H3K9 Me3 and H4Ac.

TBP-interacting protein 49b (Tip49b), a component of the INO80 chromatin-remodeling complex, is identified as a novel activating transcription factor 2 (ATF2)-interacting protein. The intact complex of Tip49b and ATF2 is dependent on ATF2 phosphorylation under various stimuli, including UV treatment and ionizing irradiation [50]. Fe65, a binding partner of Tip60, associates with chromatin under basal conditions. This association links Fe65 to DDR by affecting the level of histone H4 acetylation and assisting in the recruitment of Tip60-TRRAP to DNA damage sites [51,52] (Figure 3). The partner and localizer of the breast cancer 2 susceptibility protein (PALB2) is crucial for the repair of DNA damage by HR. PALB2, an integral component of the BRCA complex, binds directly to MRG15, while MRG15 interacts with the entire BRCA complex, which contains BRCA1, PALB2, BRCA2 and RAD51 [53,54]. Moreover, the chromatin-association motif (ChAM), an evolutionarily conserved motif in PALB2, is necessary and sufficient to mediate its chromatin association in both unperturbed and damaged cells [55]. MRG15 mediates DDR functions in chromatin by silencing the p53 signaling pathway in neural stem/progenitor cells [56].

The histone methyltransferase MMSET (also known as NSD2 or WHSC1), a newly identified histone methyltransferase [57,58], accumulates at DSBs and is induced by the γH2AX-MDC1 pathway; specifically, the interaction between the MDC1 BRCT domain and the phosphorylated Ser102 of MMST regulates the induction of H4K20 methylation on histones around DSBs, which facilitates the recruitment of 53BP1 [59]. This interaction indicates that chromatin-remodeling factors directly contribute to DNA repair by interacting with DNA damage-related proteins that may affect signaling pathways involved in DNA repair.

## 5. Modification of Chromatin-Remodeling Factors Is Linked to the DNA Damage Response

Protein modifications such as phosphorylation, methylation, and ubiquitination play a critical role in gene regulation. Acetylation and deacetylation of chromatin-remodeling factors are critical to chromatin structure and the response to DNA damage.

In unstressed cells, the interaction of Apak (ATM and p53-associated KZNF protein), a Kruppel-associated box (KRAB)-type zinc-finger protein, with p53 and KRAB-box-associated protein (KAP1) recruits ATM and HDAC1 to attenuate p53 acetylation, thereby selectively turning off the expression of pro-apoptotic genes [60]. However, in response to DNA damage, ATM is rapidly activated by autophosphorylation and mediates p53 activation through disruption of the Apak-p53 complex by phosphorylating Apak on Ser68. This disruption allows p53 to become fully activated by phosphorylation and acetylation, resulting in the activation of its target genes and apoptosis [60].

DSB repair occurs within chromatin and can be modulated by chromatin-modifying enzymes. As participants in DDR, human histone deacetylases HDAC1 and HDAC2 localize to sites of DNA damage, promoting to deacetylation of histone H3 Lys56 (H3K56) and histone H4 Lys 16 (H4K16) [61]. DSBs induced by chemotherapy and IR release HDAC1 from the complex of HDAC1, PML and HBV-core in the HBV replication cycle. During chemotherapy and radiotherapy, PML in PML-NBs could link the DNA damage response to HBV replication. Additionally, PML may interact with HBV-core and HDAC1 on the promoter of the covalently closed circular HBV DNA basal core [62]. In mammals, Sirtuin 6 (SIRT6), a mammalian homolog of the yeast Sir2 deacetylase, is required for genome instability. SIRT6 is recruited to the DSB sites and stimulates DSB repair through both NHEJ and HR [63]. SIRT6 physically links with poly [adenosine diphosphate (ADP)–ribose] polymerase 1 (PARP1) and mono-ADP-ribosylates PARP1 on lysine residue 521, thereby stimulating PARP1 poly-ADP-ribosylase activity and enhancing DSB repair under oxidative stress [63].

Tip60 also controls chromatin remodeling in response to DNA damage by protein modifications such as acetylation. Human Rvb1 and Rvb2 are highly conserved AAA^+^ ATP binding proteins that are part of various chromatin-remodeling complexes, such as Ino80, SNF2-related CBP activator protein (SRCAP), and Tip60/NuA4 complexes. The depletion of Rvb1 increases the amount and persistence of phosphorylation on chromatin-associated H2AX after the exposure of cells to UV irradiation or to DNA damage-inducing reagents, while Tip60 depletion mimics the effect of Rvb1 depletion on H2AX phosphorylation [64]. Rvb1 is required for the histone acetyltransferase (HAT) activity of the Tip60 complex, and histone H4 acetylation is required prior to the dephosphorylation of phospho-H2AX [64], implicating the Rvb1-Tip60 complex in the chromatin-remodeling response of cells after DNA damage.

The MORF4-related gene on chromosome 15 (MRG15) has been identified as a core component of the NuA4/Tip60 histone acetyltransferase complex that modifies chromatin structure in response to DNA damage such as gamma irradiation [65]. Formation of phosphorylated H2AX and 53BP1 foci is attenuated after the depletion of Mrg15 following irradiation [65]. Interestingly, sumoylation of Tip60 at lysines 430 and 451 via Ubc9 promotes Tip60 from the nucleoplasm to the promyelocytic leukemia (PML) nuclear body (NB) where it is necessary for DDR in a p53-dependent pathway [66].

The interaction of the Tip60 HAT complex with H2AX, a DNA damage response marker, increases in response to irradiation [67]. Human Tip60 promotes the acetylation-dependent ubiquitination of H2AX by Ubc13, causing H2AX release from chromatin, which facilitates chromatin reorganization following DNA damage [67]. Recently, two E3 ligases, RNF8 and RNA168 were determined to control the ubiquitination of H2A and H2AZ at damaged chromosomes in response to DNA damage signaling [68,69]. However, how these proteins are recruited to the damaged chromatin by a chromatin-remodeling factor remains unclear.

## 6. Direct Effects of Chromatin-Remodeling Factors on the Recruitment of DNA Damage Response Proteins

The chromatin-remodeling factor Tip49 recruits Rad51 to DNA damage sites. Both depletion of Tip49 and Tip48 redistribute Rad51 to chromatin and nuclear foci after DSBs and interstrand crosslinks. Moreover, reduction of Rad51 foci in Tip49 knockdown cells is not due to defective DNA damage checkpoint signaling. Sodium butyrate, an inhibitor of histone deacetylases, restored the formation of Rad51 foci in Tip49 knockdown cells, indicating that Tip49 may directly facilitate the access of the repair machinery to sites of DNA damage [70].

The INO80 complex in budding yeast is a conserved ATP-dependent nucleosome remodeler containing actin-related proteins Arp5 and Arp8. Strains lacking INO80, Arp5, or Arp8 are hypersensitive to DNA damaging agents and to DSBs induced by the HO endonuclease. The members of the INO80 complex are recruited to an HO-induced DSB, where a phosphorylated form of H2A S129 accumulates. The INO80 chromatin-remodeling complex is associated with stalled replication forks. Because INO80 has a strong impact on the steady state levels of H2A.Z acetylation, depletion of INO80 performs a boundary function by removing H2A.Z that is deposited distal to the targeted SWR-C enzyme [4,71,72].

In yeast, the Iec1 protein, an Ino80-associated and highly conserved complex, functions as a novel zinc-finger protein with similarities to the mammalian transcriptional regulator Yin Yang 1 (YY1) and other members of the GLI-Krüppel family of proteins. Iec1 decreases the response to DNA damage repair [73].

These studies indicate that protein–protein interactions between chromatin-remodeling factors and DNA damage-related proteins contribute to DDR. It is not clear which motifs of chromatin-remodeling factors are involved in the DNA damage sites, so this question requires further study.

## 7. Conclusion and Perspective

DNA methylation is a key feature in epigenetics. It is identified as CpG methylation, non-CpG methylation and hydromethylcytodine (hmC). DNA damage affects the switch of DNA methylation. Chromatin structure, in turn, is also affected by chromatin-remodeling factors, and it will be interesting to address the process of dynamic switches in response to DDR. Chromatin-remodeling factor lymphocyte specific helicase (LSH) produces DNA methylation at CpG and non-CpG sites [74]. It will be of interest to address whether and how DNA methylation links chromatin-remodeling factors in response to DDR.

Chromatin-remodeling factor binding to genomic DNA protects these underlying sequences from cleavage by DDR. Recently, a large collection of novel regulatory factor recognition motifs were discovered that are highly conserved in both sequence and function, and that exhibit cell selective occupancy patterns that closely associate with development, differentiation and pluripotency [75], demonstrating that chromatin remodeling in response to DNA damage is more complex. Chromatin forms loops and links with the bound chromatin-remodeling factors, in turn affecting gene expression, and revealing a novel function for chromatin looping [76,77]. Thus, it would be very interesting to use this technique to reveal whether chromatin-remodeling factors form more highly ordered chromatin fiber structures and whether DDR can alter the patterns of genome architecture such as chromosome looping and the enhancer or insulator function for these loci.

Changes in protein localization and abundance reveal specific patterns of functional enrichment in response to DNA damage at the global level [78]. Genomic instability is one of the most pervasive characteristics of tumors and is most likely the combined effect of DNA damage, chromatin disruption and cell cycle disruption [79]. Epigenetic disruption such as DNA methylation and production of non-coding RNAs is a characteristic of human cancer [78,80]. The DNA damage response contributes to tumor prevention in the early stages of human tumorigenesis by causing cell-cycle blockade, cell differentiation and apoptosis [1,81]. Non-coding RNAs directly control DDR activation at sites of DNA damage [82]. The cancer epigenome undergoes global changes in the patterns of DNA methylation, histone modification and chromatin-remodeling factors, all of which play important roles in cancer initiation and progression. Obviously, abnormal epigenetic changes to chromatin regulatory complexes will disrupt the DNA damage repair process during carcinogenesis. DNA damage response signaling pathways affected by chromatin-remodeling factors are therefore strong candidates for drug targets. Additionally, a better understanding of DDR will expand our knowledge of cancer development and contribute to efforts to treat the disease.
